# Multi-Drone Cooperation for Improved LiDAR-Based Mapping

**DOI:** 10.3390/s24103014

**Published:** 2024-05-09

**Authors:** Flavia Causa, Roberto Opromolla, Giancarmine Fasano

**Affiliations:** Department of Industrial Engineering, University of Naples “Federico II”, 80125 Naples, Italy; roberto.opromolla@unina.it (R.O.); giancarmine.fasano@unina.it (G.F.)

**Keywords:** LiDAR mapping, cooperative UAVs, cooperative navigation, cooperative mapping, georeferencing accuracy, point-density prediction, attitude requirements

## Abstract

This paper focuses on mission planning and cooperative navigation algorithms for multi-drone systems aimed at LiDAR-based mapping. It aims at demonstrating how multi-UAV cooperation can be used to fulfill LiDAR data georeferencing accuracy requirements, as well as to improve data collection capabilities, e.g., increasing coverage per unit time and point cloud density. These goals are achieved by exploiting the CDGNSS/Vision paradigm and properly defining the formation geometry and the UAV trajectories. The paper provides analytical tools to estimate point density considering different types of scanning LIDAR and to define attitude/pointing requirements. These tools are then used to support centralized cooperation-aware mission planning aimed at complete coverage for different target geometries. The validity of the proposed framework is demonstrated through numerical simulations considering a formation of three vehicles tasked with a powerline inspection mission. The results show that cooperative navigation allows for the reduction of angular and positioning estimation uncertainties, which results in a georeferencing error reduction of an order of magnitude and equal to 16.7 cm in the considered case.

## 1. Introduction

Unmanned aerial vehicle (UAV)’s flexibility and adaptiveness to several environments have stimulated their usage in multiple application domains to execute tasks which required humans or manned vehicles in the past. Besides these applications, new missions are expected to be carried out by UAVs in the next few years, including urban transportation and delivery within the Advanced Air Mobility framework [1]. As a consequence, the UAV market is constantly growing and is expected to be worth hundreds of billions of dollars at the end of this decade. Forty-five percent of the current UAV market [2] includes infrastructure inspection and monitoring, and several scientific and industrial products have been developed to make UAVs and their embarked payloads more and more effective in performing these missions [3]. Electro-optical sensors, like passive cameras or Light Detection and Ranging (LiDAR) instruments, are typically used as mapping payloads, i.e., to collect data suitable for creating a georeferenced 3D map of the environment, in several scenarios [4], including the inspection of powerlines [5], bridges [6], and ground terrain [7,8]. If LiDAR is used, the 3D map generation task requires an accurate UAV navigation solution to be retrieved, as well as that the platform path is properly defined to solve the complete coverage problem (CCP) of the area or object to inspect. 

Concerning the first point, indirect or direct georeferencing approaches can be adopted depending on whether the payload data (i.e., LiDAR-generated points) are also used or not in the UAV trajectory estimation process. Indirect georeferencing with omnidirectional LiDAR has been carried out in [9], using loop closure to avoid odometry divergence, while reference [10] has proposed a tight integration of non-repetitive scanning LiDAR measurements within a Kalman filter to simultaneously estimate both the vehicle trajectory and the map of the environment. Direct LIDAR georeferencing [11,12], instead, requires that an estimate of the UAV trajectory is obtained before the payload data are processed. In particular, both accurate positioning and attitude states should be computed to attain a high-quality map product, i.e., characterized at most by a submeter error level. With regard to positioning, centimeter-level accuracy can be obtained with commercial GNSS receivers thanks to advanced processing techniques [13], like Precise Point Positioning (PPP) in a stand alone configuration or carrier-phase differential GNSS (CDGNSS) and Real Time Kinematic (RTK) when a single, or a network, of ground stations is used. With regard to attitude determination, instead, sub degree-level accuracy can be achieved by using a high cost/high performance inertial measurements unit (IMU) integrated with a high accuracy GNSS solution. A less costly alternative consists of relying on additional aids coming from dedicated onboard sensors and/or external sources of information. As an example, a multi-antenna GNSS approach was exploited in [14] for a precise estimation of the platform attitude, obtaining sub decimeter mapping accuracy. Cooperative information sources have been also widely employed in mapping applications. A formation composed of a UAV and a UGV has been adopted in [15] to enhance navigation performance for a powerline monitoring application; specifically, it relies on relative positioning measurements retrieved with a camera mounted on the UAV, which identifies an April tag attached to the UGV. A technique for accurate attitude estimation of a chief UAV using one or more cooperative flying deputies has been recently proposed by the authors in [16]. It exploits the fact that highly accurate attitude information independent from the commonly used instruments onboard the UAVs (i.e., IMU and magnetometers) can be obtained by combining measurements of the relative chief-to-deputy line of sight (LOS) retrieved in the local navigation frame (e.g., north-east-down, NED) and in the body frame (BRF). This approach is referred to as CDGNSS/Vision since the LOS measurements in NED can be inferred from very precise CDGNSS baseline information, while the LOS measurements in BRF are retrieved by converting visual detections from camera to body coordinates (this requires either the camera mounting parameters to be determined thanks to an extrinsic calibration [17], in the case of strapdown installation, or the knowledge of gimbal angle information if a gimbaled installation is envisaged). The quality of the attitude solution provided by this CDGNSS/visual approach is strongly dependent on the geometry of the formation of the cooperative flying vehicles. 

As far as the coverage problem is concerned, several works in the open literature have proposed general or specific solutions, i.e., tailored to the structure of interest. A comprehensive overview of the CCP dealing with surveying an area from the top can be found in [18]. Other works have also proposed techniques for inspecting generic 3D objects [19], buildings [20], or bridges [21], which do not require nadir-looking-mounted sensors. A generalized solution for inspecting both horizontal or non-horizontal surfaces with a single LiDAR-equipped UAV is proposed by [22]. When multiple UAVs are accounted for, the Cooperative CCP (C-CCP) can be addressed by segmenting the area to inspect between the platforms. Techniques have been developed for the cooperative mapping of both horizontal areas (using nadir-looking sensors [23,24]) and 3D objects [25]. In this regard, the design of the multi-UAV system is commonly aimed at maximizing the coverage. This is typically conducted by keeping the vehicles far from each other, which can, however, hinder the possibility to exploit relative sensing and information exchange. 

In this framework, this paper investigates the advantages of using a cooperative formation of UAVs for direct LIDAR-based georeferencing. Indeed, UAV cooperation can be exploited both to improve trajectory reconstruction, thus making the navigation error compliant with georeferencing accuracy requirements, and/or to provide improved data collection capabilities. In the first case, the mapping role is entrusted to a single LiDAR-equipped vehicle in the cooperative formation. Conversely, when cooperation is used as a multi-objective instrument, i.e., both for navigation and data collection, all the vehicles of the formation should be equipped with LiDAR. 

The main contribution of this paper consists of providing a methodological framework to define cooperative UAV paths fulfilling improved navigation accuracy and data collection capabilities in LiDAR-based mapping applications, which also includes the following:

- An analytical expression for determining the LiDAR point density, considering different scanning LiDAR types (i.e., with non-repetitive or repetitive scanning pattern) and mounting configurations and accounting for the distance from the object to inspect. The accuracy of these analytical models is demonstrated by comparing the analytical predictions with point densities computed using a LiDAR simulator.
- A methodology to find the path requirements aiming at solving the CCP (applicable if the LIDAR is embarked by a single vehicle in the UAV formation) and the C-CCP (applicable if all the cooperative flying vehicles share the mapping task). It allows for the identification of the preferred LiDAR mounting configuration depending on the target to inspect and enables one to obtain a path solution which complies with the required point density.
- An analytical model for predicting the attitude accuracy needed to fulfill an assigned georeferencing requirement.
- A metric for predicting the attitude error obtained using a cooperative formation, as a function of its geometry is formulated by extending the geDOP concept introduced in [26].

The remainder of the paper is organized as follows. An analytical formulation for LiDAR-based point cloud density is derived in Section 2. The path requirements (velocity and distance from the target to be inspected) to solve both the CCP and C-CCP are reported in Section 3. Section 4 connects georeferencing accuracy requirements to attitude accuracy. Section 5 recalls the cooperative navigation algorithm used to improve the navigation accuracy and defines the innovative metric for predicting the attitude error given the formation geometry. Section 6 leverages on the tools defined in the previous section to define the proper UAV paths (for the case of a three-UAV formation) if the mapping task is entrusted either to a single UAV or to the entire formation. Based on numerical simulations, Section 7 demonstrates the validity of the proposed approach by assessing the UAV trajectory estimation accuracy and the corresponding georeferencing error in both the above-mentioned cases focusing on a powerline inspection task. Finally, Section 8 draws conclusive remarks.

## 2. Analytical Model for LiDAR Point Density

Point density is a fundamental parameter characterizing the goodness of data retrieval from the observed scene. The achievable point density depends on LiDAR specifics, platform velocity (whose module is *v*), and distance from the object (*d*) estimated along the boresight direction. In the following, LiDAR point density is derived considering a flat surface at distance *d* from the sensor both for an omnidirectional scanning pattern, e.g., Velodyne-like [27], and a non-repetitive scanning pattern, e.g., Livox-like [28]. In the former case, *n* rays, equally spaced in the vertical direction, are used to scan a 360° horizontal field of view (FOV) with a certain rate (*f*): the resulting scanning pattern instead covers a limited FOV in the vertical direction. In the latter case, a fast-moving ray is rotated to follow a floral-like pattern while covering a circular FOV. 

### 2.1. Static Point Density

**Figure 1:**
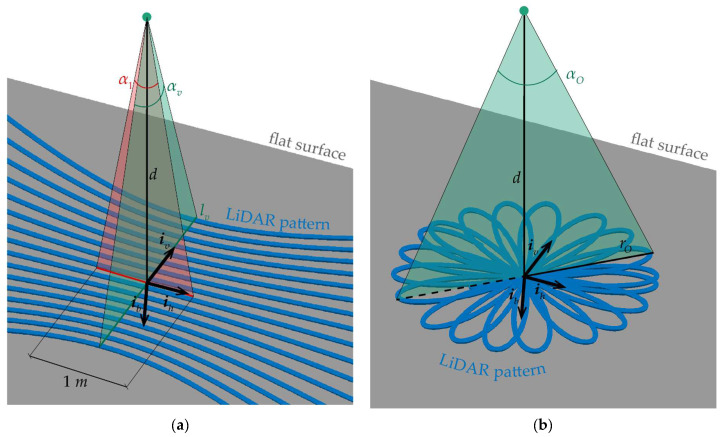
Points acquired by a static scanner on a plane with distance *d* from the sensor with (**a**) omnidirectional scanning pattern and (**b**) non-repetitive scanning pattern.

The static point density *ω*_s_ is the one that a steady LiDAR would produce. It is derived by counting the points for unit area in a static configuration. The points retrieved on a flat surface at distance *d* are reported in Figure 1a,b for omnidirectional and non-repetitive scanning patterns, respectively. In both figures, the two cross-boresight directions of a sensor-fixed reference frame, i.e., ***i****_v_* (vertical) and ***i****_h_* (horizontal), are reported along with the boresight axis, i.e., ***i****_b_*. For the sake of figure readability, the triad has been centered in the point where the boresight axis crosses the flat plane. The following expression holds true, ***i****_h_* = ***i****_b_* × ***i****_v_*. 

For omnidirectional scanners, the static point density (*ω_s_*_,omni_) is derived while focusing on a square area of 1 m^2^ around the boresight, and it can be defined as the product between two linear densities, i.e., the vertical *ω_s,v_* and horizontal *ω_s,h_* ones, as follows:

(1) $$\omega_{s,\mathrm{omni}}=\omega_{s,v}\omega_{s,h},$$

The value of *ω_s,v_* can be computed dividing the number of stripes (*n*) by the distance covered by the rays along ***i****_v_* at boresight, which is referred to as *l_v_* in Figure 1a, as follows

(2) $$\omega_{s,v}=\frac{n}{l_{v}}=\frac{n}{2d\tan\left(\frac{\alpha_{v}}{2}\right)},$$

where *α_v_* is the vertical FOV, reported in Figure 1a. It is important to remark that Equation (2) is based on a rectangular approximation of the lidar footprint which holds true around the boresight. The horizontal point density is defined by counting the points contained in a 1 m width along ***i****_h_*. This quantity corresponds to the ratio between the angle underlining the 1 m width, i.e., α_1_ shown in Figure 1, and the horizontal resolution of the LiDAR, (*ε_h_*):

(3) $$\omega_{s,h}=\frac{\alpha_{1}}{\varepsilon_{h}}=\frac{2\tan^{-1}\left(\frac{1}{2d}\right)}{\varepsilon_{h}},$$

If a non-repetitive scanning pattern is used, a fixed angular resolution of the scanner cannot be considered, and the static point density can be derived accounting for the point rate (number of points per second, i.e., ${\dot{n}}_{O}$) and setting an integration time (*T_O_*). The number of points collected during *T_O_* must be divided by the area of the circle with radius *r_O_*, depicted in Figure 1b. Thus, the point density is as follows:

(4) $$\omega_{s,\mathrm{nrep}}=\frac{{\dot{n}}_{O}T_{O}}{\pi r_{O}^{2}}=\frac{{\dot{n}}_{O}T_{O}}{\pi {\left(d\tan\left(\frac{\alpha_{O}}{2}\right)\right)}^{2}},$$

where *α_O_* is the size of the non-repetitive scanner’s circular FOV.

### 2.2. Moving Point Density

When the motion of the platform is accounted for, the norm of the velocity plays a fundamental role in the definition of the point density. LiDARs are usually moved in the plane orthogonal to the boresight. While for non-repetitive scanners the estimation of the point density is independent from the direction taken in this plane, omnidirectional scanners benefit from being moved along ***i****_v_*. Therefore, the LIDAR motion direction is assumed to be parallel to ***i****_v_* in the following discussion. 

The omnidirectional point density, i.e., *ω*_omni_, can be still derived with Equation (1), but considering the moving vertical (*ω_v_*) and horizontal (*ω_h_*) densities. Since the motion occurs in the direction parallel to ***i****_v_*, the horizontal density is equal to the static case, i.e., *ω_h_* = *ω_s,h_*. Conversely, *ω_v_* can be computed as follows:

(5) $$\begin{array}{c} \omega_{\mathrm{omni}}=\omega_{v}\omega_{s,h}\\ \omega_{v}=\frac{l_{v}/v}{1/f}\omega_{s,v} \end{array},$$

The coefficient which scales *ω_s,v_* to its moving counterpart is the ratio between the time the vehicle needs to cover a distance equal to *l_v_* and the scanning period. 

**Figure 2:**
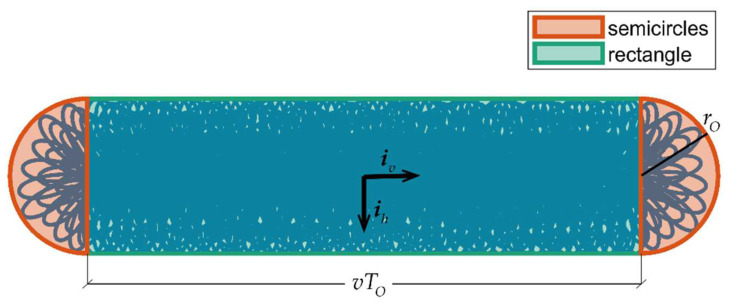
Area on ground covered by a non-repetitive scanning LIDAR with velocity *v* on a plane with distance *d* from the sensor.

The moving point density for the non-repetitive scanning pattern can be retrieved with the same logic as for Equation (4), considering the whole area covered by the scanner during *T*_O_. This quantity is equal to the area of the previously considered circle plus the area of the rectangle defined moving the circle diameter along the direction of motion for *T*_O_ seconds. The rectangle, which is depicted in Figure 2, has one side with length equal to the circle diameter, i.e., 2*r_O_*, and the other whose length is equal to the distance covered by the vehicle in *T*_O_ seconds, i.e., *vT*_O_. Hence, in analogy with Equation (4), the dynamic point density can be written as follows:

(6) $$\omega_{\mathrm{nrep}}=\frac{{\dot{n}}_{O}T_{O}}{\pi r_{O}^{2}+vT_{O}\cdot 2r_{O}}=\frac{{\dot{n}}_{O}T_{O}}{\pi {\left(d\tan\left(\frac{\alpha_{O}}{2}\right)\right)}^{2}+vT_{O}\cdot 2d\tan\left(\frac{\alpha_{O}}{2}\right)}.$$

### 2.3. Analytical Model Validation

The analytical models derived in the previous subsection have been validated by comparing the predicted point density (*ω*) with that obtained by means of numerical simulations ($\overset{⌣}{\omega }$), carried out in the MATLAB environment using ray tracing functionalities. The Velodyne Puck 16^TM^ and the Livox mid-40^TM^, whose parameters are reported in Table 1, have been selected as omnidirectional and non-repetitive scanners, respectively. 

**Figure 3:**
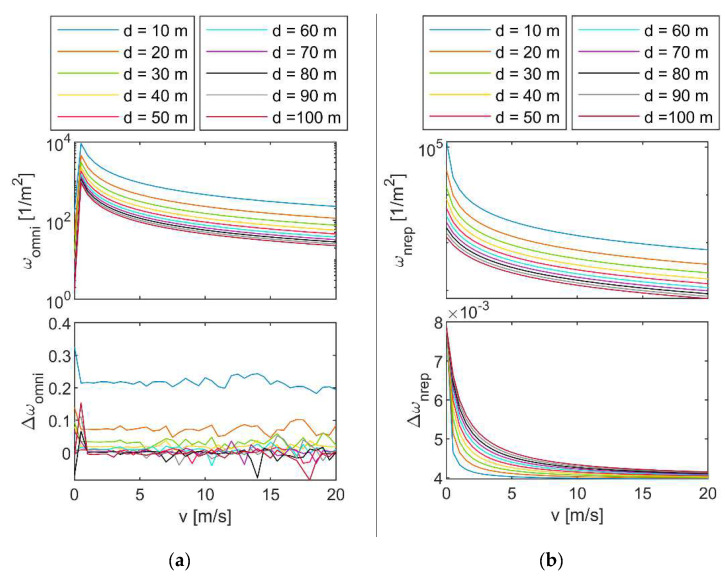
Point density (*ω*) and its estimation error (Δ*ω*) obtained using LiDAR moving at velocity *v* at distance *d* from a plane with (**a**) omnidirectional and (**b**) non-repetitive scanning patterns.

Simulated point density is obtained for omnidirectional scanners by using the MATLAB-based LiDAR sensor simulation function and counting the points fitted in a rectangle centered around the scanner’s boresight direction when the sensor is at the center of its trajectory. Conversely, for the non-repetitive scanners, $\overset{⌣}{\omega }$ is computed as the ratio between the total number of points collected along the defined trajectory (as obtained using the scanning pattern retrieved from the Livox simulator [29]) and the area covered by these points. This latter quantity is defined by substituting *r_O_* with the distance on ground of the farthest simulated point in the ***i****_h_* direction in Figure 2. The relative prediction errors, i.e., $\Delta \omega =\left(\omega -\overset{⌣}{\omega }\right)/\omega$, as well as the values of *ω*, are reported in Figure 3a and b, for Velodyne and Livox scanners, respectively, as a function *v* and *d*. An integration time (*T*_O_) of 60 s has been assumed for the non-repetitive scanner. 

While for the non-repetitive scan pattern, the point density monotonically reduces with larger *d* and *v*, when an omnidirectional LiDAR is used the point density reaches its minimum value when *v* = 0, showing a sudden increase when the platform starts moving, followed by a smoother decreasing trend at increasing ***v***. The plots show that the analytical models produce a very small error. A relatively larger error is obtained, especially at lower altitudes in the omnidirectional case (reaching up to 30% error when *d* = 10 m). At these altitudes, the analytical model formulated herein outputs a higher point density than the expected one. This behavior manly depends on the fact that when *d* reduces, the projection of the scanner’s ray on the flat surface assumes a shape which deviates from the rectangle, which has been assumed in the analytical formulation. However, this difference is negligible, producing an error smaller than 5% when *d* > 30 m. As reported in Figure 3, non-repetitive scanners allow for increasing the point density of an order of magnitude, even if characterized by a smaller value of ${\dot{n}}_{O}$. This is due to the smaller FOV in which the points are collected.

**Table 1:** Specifics of the omnidirectional and non-repetitive scanners.

Parameter	Omnidirectional Scanner	Non-Repetitive Scanner
Type	Velodyne Puck	Livox mid-40
Vertical FOV, $\alpha_{v}$ [deg]	30	N/A
Horizontal FOV, $\alpha_{h}$ [deg]	360	N/A
Circular FOV, $\alpha_{O}$ [deg]	N/A	38.4
Vertical Resolution, $\varepsilon_{v}$ [deg]	2	N/A
Horizontal Resolution, $\varepsilon_{h}$ [deg]	0.1 ^1^	N/A
Scanning frequency, *f* [Hz]	5 ^1^	N/A
Number of parallel rays, *n*	16	N/A
Maximum Range, *R*_max_ [m]	100	90–260
Points per second, ${\dot{n}}_{O}$ [1/s]	300,000	100,000

^1^ Velodyne Puck frequency can be set from 5 to 20 Hz, which affects the horizontal resolution, producing an increase from 0.1° to 0.4°.

## 3. Single Aircraft and Cooperative Complete Coverage Problem Solution Approaches

The CCP requires the definition of UAVs’ paths which allow full coverage of an area. Depending on the typology of target area to be mapped, different LiDAR mounting configuration, and thus trajectory design approaches, must be adopted. Specifically, LiDAR could be mounted with a nadir-looking or a frontal-looking configuration, or with a generic off-nadir angle (between 0° and 90°), allowing the collection of both nadir and lateral points. The first configuration is suitable for inspection of large areas, e.g., if a map for a wide urban environment is requested. In such case, a small point density is expected in the lateral faces of the buildings with respect to the horizontal ones, i.e., roofs [22]. Nadir-looking LiDARs have also been extensively used in powerline inspection missions. On the other hand, lateral LiDAR installation is useful when the target is a prism-like object, such as a building, around which the UAV should fly over a spiral-like trajectory. In both cases, a zig-zag trajectory, built up from parallel lines with constant distance, can be used to fulfill the CCP [22]. 

Path definition requires the identification of the separation between the lines of zig-zag paths, as well as of the velocity and distance from the target to be inspected. These parameters must be selected depending on the installation of the LiDAR on the UAV, whose orientation is defined with respect to the Body Reference Frame (BRF) for which a forward-right-down convention is adopted. The *j*-th axis (*j* = 1, 2, 3) of the BRF is referred to as ***b****_j_* in the following. Since the point density benefits from moving the LiDAR along ***i****_v_*, LiDAR is commonly installed on a UAV with the vertical axis aligned to the motion direction, i.e., $i_{v}\equiv b_{1}$, while the boresight axis lies in the ***b***_2_–***b***_3_ plane (which thus represents the horizontal scan plane) and its angle from the ***b***_2_ direction is referred to as squint angle, i.e., *γ* ∈ [0,90°], so that

(7) $$\begin{array}{l} i_{v}\equiv b_{1}\\ i_{b}\equiv b_{2}\cos\gamma +b_{3}\sin\gamma \\ i_{h}\equiv -b_{2}\sin\gamma +b_{3}\cos\gamma \end{array}.$$

**Figure 4:**
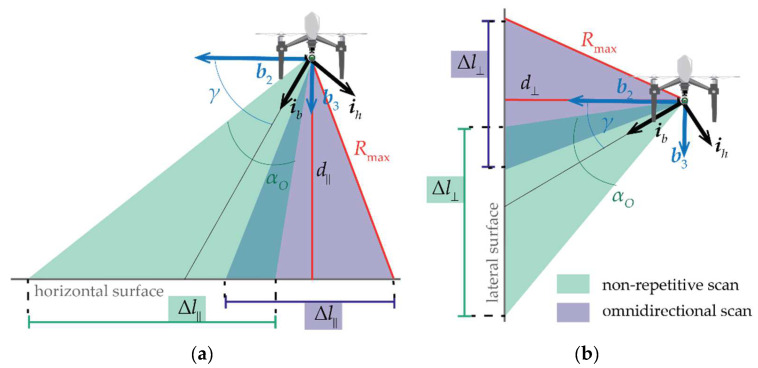
LIDAR footprint on the *b*_2_–*b*_3_ plane for omnidirectional and non-repetitive scan patterns. (**a**) Nadir-looking geometry, i.e., viewed surface orthogonal to *b*_3_; (**b**) lateral-looking geometry, i.e., viewed surface orthogonal to *b*_2_. Please note that for the representation of the footprint of a non-repetitive scan pattern, the limitation due to the maximum LIDAR measurable range (*R_max_*) is not considered.

The LiDAR footprint (Δ*l*_•_) and the distance from the surface to inspect (*d*_•_) are the lengths of two segments belonging to the horizontal scan plane (as shown in Figure 4) and can be defined with respect to ***b***_2_ (i.e., the target area is a lateral surface orthogonal to the ground plane) or ***b***_3_ (i.e., the target area is a horizontal surface parallel to the ground plane) by setting the pedix • with the symbol ⟂ and ‖, respectively. Clearly, in the case of a squinted LIDAR configuration (i.e., 0° < *γ* < 90°), both Δ*l*_‖_ and Δ*l*_⟂_ can be non-zero for both lateral and horizontal surface inspection. 

As far as *d*_•_ is concerned, its maximum allowable value should be consistent with the maximum range of the LiDAR referred to as *R*_max_. To ensure that all the LIDAR rays can produce valid measurements, *d*_•_ should be smaller than cos(α_O_/2)*R*_max_ and cos(α*_v_*/2)*R*_max_ in the non-repetitive or omnidirectional case, respectively. Considering the values of *R*_max_ reported in Table 1, Figure 3 shows that up to these distances, the point density of the two analyzed LiDAR types at typical cruise velocities of small UAS (i.e., 8 m/s) is higher than 54 points/m^2^, resulting in an average spatial resolution of about 0.14 m. Since this value is satisfactory for mapping applications [5], the LiDAR can assume any distance from the surface, which is compliant with the maximum range by reducing the velocity if a further increase in point density is required. Although small distances from the target are preferred because of the increased point density, operating at higher distances enlarges the footprint of the sensor, allowing coverage of a larger area in a reduced time. In addition, the advantage of operating at a larger distance becomes clear in missions such as urban inspection, where the UAV altitude (and therefore the lidar-to-ground distance) must be kept high to ensure an adequate separation between the UAV and the obstacles, while still guaranteeing satisfactory resolution in ground terrain mapping. This approach is also applied in powerline inspection, where magnetic interference suggests operating at an altitude which is significantly higher than the powerline (and depends on its voltage [30]), obtaining a terrain separation in the order of about 65 m when a high-voltage 275 kV powerline is considered. 

In the omnidirectional LiDAR case, Δ*l*_•_ is independent from *γ* and varies as a function of *d*_•_, while being limited by the sensor’s maximum measurable range, i.e., *R_max_*, as follows:

(8) $$\Delta l_{•}=2\sqrt{R_{\max}^{2}-d_{•}^{2}},$$

Conversely, if a non-repetitive scanning pattern is considered, Δ*l*_‖_ and Δ*l*_⟂_ values are also limited by *γ* and can be expressed as follows:

(9) $$\begin{array}{l} \Delta l_{∥}=\{\begin{array}{ll} 0, & \gamma <\sin^{-1}\left(\frac{d_{∥}}{R_{\max}}\right)-\frac{\alpha_{O}}{2}\\ \sqrt{R_{\max}^{2}-d_{∥}^{2}}-d_{∥}\tan\left(\frac{\pi }{2}-\gamma -\frac{\alpha_{O}}{2}\right), & \sin^{-1}\left(\frac{d_{∥}}{R_{\max}}\right)-\frac{\alpha_{O}}{2}<\gamma <\sin^{-1}\left(\frac{d_{∥}}{R_{\max}}\right)+\frac{\alpha_{O}}{2}\\ d_{∥}\left(\tan\left(\frac{\pi }{2}-\gamma +\frac{\alpha_{O}}{2}\right)-\tan\left(\frac{\pi }{2}-\gamma -\frac{\alpha_{O}}{2}\right)\right), & \gamma >\sin^{-1}\left(\frac{d_{∥}}{R_{\max}}\right)+\frac{\alpha_{O}}{2} \end{array}\\ \Delta l_{⊥}=\{\begin{array}{llll} 0, & & & \gamma >\cos^{-1}\left(\frac{d_{⊥}}{R_{\max}}\right)+\frac{\alpha_{O}}{2}\\ \sqrt{R_{\max}^{2}-d_{⊥}^{2}}-d_{⊥}\tan\left(\gamma -\frac{\alpha_{O}}{2}\right), & & & \cos^{-1}\left(\frac{d_{⊥}}{R_{\max}}\right)-\frac{\alpha_{O}}{2}<\gamma <\cos^{-1}\left(\frac{d_{⊥}}{R_{\max}}\right)+\frac{\alpha_{O}}{2}\\ d_{⊥}\left(\tan\left(\gamma +\frac{\alpha_{O}}{2}\right)-\tan\left(\gamma -\frac{\alpha_{O}}{2}\right)\right), & & & \gamma <\cos^{-1}\left(\frac{d_{⊥}}{R_{\max}}\right)-\frac{\alpha_{O}}{2} \end{array} \end{array}.$$

It is intuitive that a small squint angle produces a null value for Δ*l*_‖_. Conversely, if *γ* approaches 90°, no measurements are retrieved from lateral surfaces. So, in order to obtain finite values for both Δ*l*_‖_ and Δ*l*_⟂_, an intermediate value of *γ* (e.g., =45°) should be selected. Instead, missions which inspect either surface from the top or sideward are suggested to be performed with *γ* = 90° or *γ* = 0°, respectively. 

### 3.1. CCP Problem for a Single UAV

As mentioned before, a zig-zag path is one of the most efficient solutions to solve the CCP. Such path lies on a plane parallel to the object to inspect and is placed at a constant distance from it: it is composed of parallel lines with an interline separation computed as $\Delta s_{•}=\Delta l_{•}\left(1-m_{s}\right)$ [31], where Δ*l*_•_ can be either Δ*l*_‖_ or Δ*l*_⟂_ for horizontal and lateral surface inspection, respectively, while *m_s_* is a safety margin (ranging between 0 and 1) that produces overlaps (also referred to as sidelaps) between the areas observed from two subsequent parallel lines. 

**Figure 5:**
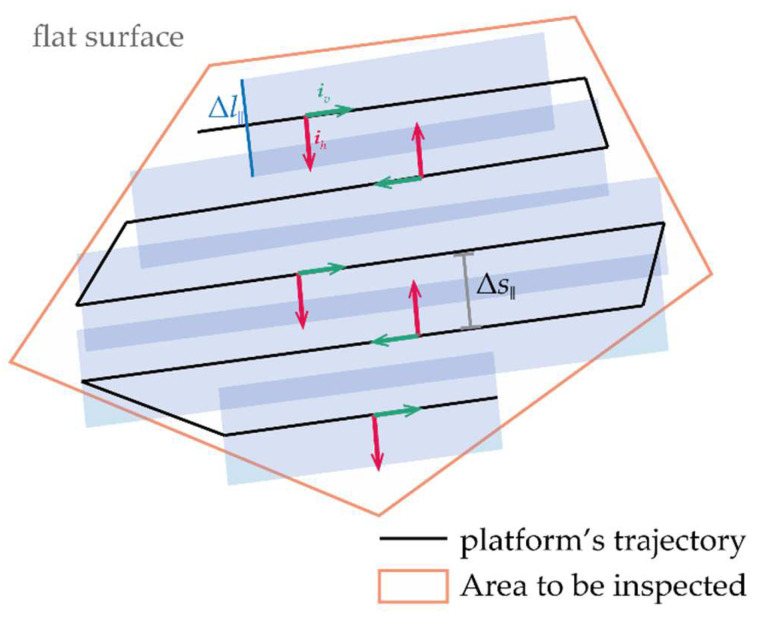
CCP solution for mapping a horizontal surface: example of zig-zag path.

If a ground (horizontal) area has to be mapped, the UAV should cover a generic convex polygon flying at a constant altitude *d*_‖_ from the terrain with the LiDAR pointed downwards (*γ* = 90°). Hence, Δ*l*_⟂_ is 0, while Δ*l*_‖_ (defined using LiDAR parameters and *d*_‖_) is used to compute Δ*s*. The direction of the parallel lines on the local horizontal plane, i.e., the motion direction, can be selected as the one minimizing the number of turns, as they cause UAV deceleration [31]. An example of this procedure showing the resulting path on the local horizontal plane is reported in Figure 5. When the mapping area can be approximated to a rectangle presenting one direction much larger than the other, as in the powerline case, the UAV could cover the whole area with a single passage if a *d*_‖_ providing a Δ*l*_‖_ larger than the width of the shorter rectangle’s side exists and is compliant with airspace altitude limits. In this case, the point density can be made compliant with the required one by reducing the UAV’s velocity. 

**Figure 6:**
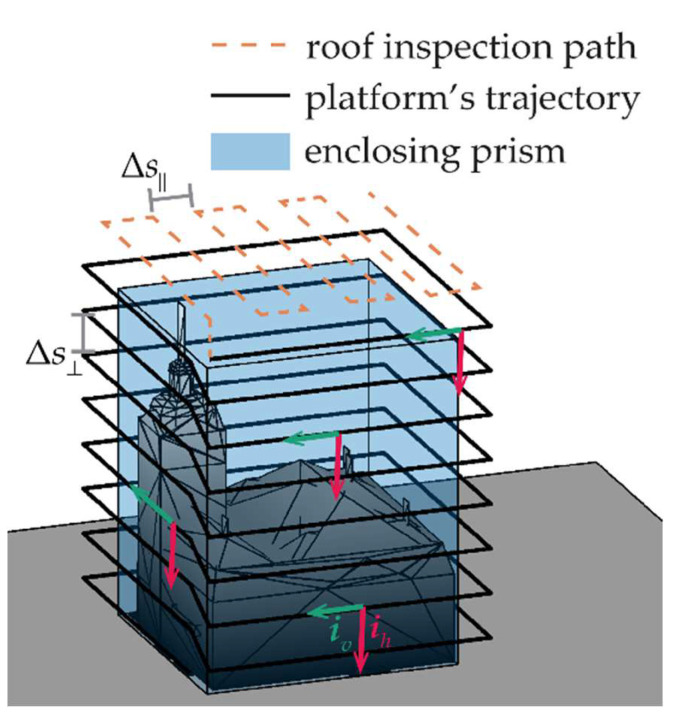
CCP solution for building inspection. The building can be enclosed in a prism whose shape is reported by the blue 3D figure. Two zig-zag paths are defined to inspect the lateral surfaces (solid black line) and the roof (dashed orange line).

The same zig-zag motion solution can be applied to the case of lateral surface mapping, where, as suggested by [22], the area to be mapped can be approximated as a prism (or a cylinder) enclosing the object to map. This shape can be unfolded as a plane to define the number of parallel lines the UAV has to fly on, assuming the UAV has to keep a constant lateral separation from the prism’s face, i.e., *d*_⟂_. In this case, *γ* can be set to 0°. An example of a building with an enclosing prism is reported in Figure 6, along with the path of the UAV. Differently from reference [22], in which the direction of the parallel lines is defined to be aligned with the local vertical, a solution based on horizontal parallel lines is proposed in this paper. This strategy allows us to reduce the energy consumption of the UAV, which is stressed by continuous ascending and descending maneuvers, as in [22]. It is important to remark that the lateral trajectory (reported with a solid line in Figure 6) does not allow for a complete inspection of the building, which should also include taking points from the roof. To this aim, a trajectory like the one reported by the orange dashed line should be followed by the UAV. However, due to the LiDAR installation, which is fixed in the body frame, only omnidirectional LiDARs which have a 360° FOV around ***i****_v_* would be able to take points from the roof surface. Conversely, when a non-repetitive scanner is used, due to its limited horizontal FOV, either a squinted or a pointable installation of the sensor could be envisaged to fulfill both the lateral and the downward mapping capability. However, the latter may be undesirable due to the variable installation matrix for the LiDAR. The trajectory reported in Figure 6 is obtained by defining vertical and lateral separation of the lines, i.e., Δ*s*_⟂_ and Δ*s*_‖_ as a function of the lateral and nadiral footprint of the sensors, i.e., Δ*l*_⟂_ and Δ*l*_‖_, which in the case of a squinted LiDAR installation should be both non null.

A combined path solution is instead requested when the object to inspect has both large horizontal extension and lateral details to be captured. In this case, horizontal and lateral inspection methods can be used in a two-step inspection solution envisaging first the nadiral inspection of the area and then the collection of lateral points where the density provided by the nadir-looking inspection is smaller than the required one.

### 3.2. Cooperative CCP 

**Figure 7:**
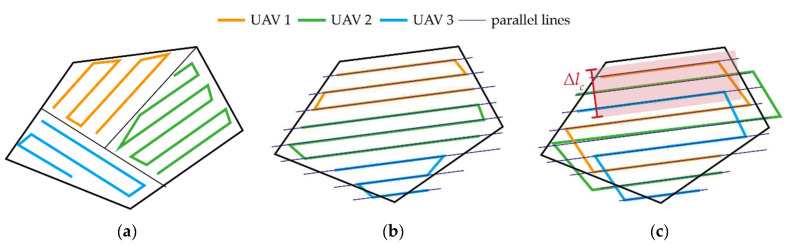
Zig-zag path solutions to the C-CCP problem: (**a**) area segmentation, (**b**) parallel independent trajectories, and (**c**) parallel cooperative trajectories.

The adoption of multi-UAV systems and swarms has been recently spread in the open literature to overcome the limits of the single vehicle, i.e., reducing mission time and/or improving mission performance. When a multi-drone system is exploited for a coverage mission which foresees a zig-zag path, the C-CCP problem can be solved by dividing the area to explore among the UAVs. Three approaches are possible in this respect, whose graphical representation is reported in Figure 7. In this case, the underlying assumption is that all the UAVs are identical, and they embark the same mapping payload, thus obtaining the same footprint, i.e., Δ*l*_•_. The first approach (Figure 7a) has been proposed in [24] and includes segmenting the area to explore in a number of polygons equal to the UAV number. For each polygon, the CCP problem is solved assuming that each UAV operates independently. Approaches (b) and (c), instead, involve the definition of parallel lines needed to cover the area as if a single UAV was operating. Once the lines have been defined, they can be assigned to each UAV by grouping (b) consecutive lines or (c) selecting adjacent lines for the vehicles, as proposed by [32], in order to make them explore the area in a close formation. Different from the other two approaches, the technique reported in Figure 7c allows the vehicles to communicate during the mission, by keeping them closer and potentially enabling the exploitation of cooperative navigation techniques. In this case, the length of the formation footprint defined over the surface to inspect and in the direction orthogonal to the motion is referred to as Δ*l_c_* (which is also reported in Figure 7c) and is equal to

(10) $$\Delta l_{c}=\left(n_{c}-1\right)\Delta s_{•}+\Delta l_{•}.$$

When cooperation is needed for purposes other than data collection (as in the cooperative navigation case), it may occur that the number of cooperative vehicles is greater than the one required for data collection aims only, thus making Δ*l_c_* potentially larger than the width of the area to map. To fully exploit the advantage of formation for mapping aid, Δ*l_c_* could be scaled down to fit the area to inspect by reducing the distance from the surface and/or the spacing between the platforms. In both cases, denser point clouds can be obtained, thus potentially enabling a velocity increase to reduce the mission time. Clearly, an increase in point density is only obtained in the area where the footprints overlap. To have a homogenous increase in the point density (which enables velocity increase), the distance between the platforms must be smaller than Δ*l*_•_/2. However, since the point density of the LiDAR reduces while going far from the boresight, especially for wide horizontal FOV (as the omnidirectional one), a much shorter separation than Δ*l*_•_/2 is suggested to enable velocity increase. 

## 4. Pointing Error and Georeferencing Accuracy

The overall georeferencing error Δ*x_req_* is the difference between the true (real-world) coordinates of a generic point on the target to be mapped and those estimated by the georeferencing process. It depends on both positioning and attitude errors of the platform performing the mapping mission. Therefore, two components can be distinguished, namely Δ*x_p_* and Δ*x*_Φ_, characterizing the positioning and attitude contribution, respectively. Using high-accuracy GNSS products, Δ*x_p_* can be limited to a few centimeters. Instead, Δ*x_Φ_* can be connected to the attitude/pointing error of the platform (ΔΦ) using at first order:

(11) $$\Delta \Phi =2\tan^{-1}\frac{\Delta x_{\Phi }}{2d}$$

where *d* is recalled being the estimated distance between the object to inspect and the sensor along the boresight direction. Clearly, if a requirement is assigned on the overall georeferencing accuracy and accounting for the positioning contribution, Equation (11) can be used to set a constraint on the maximum allowable pointing error (ΔΦ*_req_*), which decreases when the distance from the object increases. Specifically, sub-degree accuracy is requested to keep the overall georeferencing error in the order of few tens of centimeters for common operating distances (10–100 m). Such result can be obtained either by embarking high-performance inertial sensors [33] and exploiting sensor fusion with high-accuracy GNSS positioning (whose performance degrades in case of low-speed flight and hovering) or using cooperative solutions based on CDGNSS/visual techniques [16]. If the latter solution is adopted, the theoretical angular accuracy for heading, pitch, and roll angles expected by the use of CDGNSS/visual techniques (i.e., *ε_heading_* and *ε_pitch,roll_*) is derived for a quasi-horizontal formation in [16] (i.e., chief and deputy are on the same plane) and can be expressed as

(12) $$\begin{array}{l} \varepsilon_{heading}=\sqrt{{\left(2\tan^{-1}\frac{\sigma_{CDGNSS,h}}{2\sqrt{2}r}\right)}^{2}+\sigma_{cam}^{2}}\\ \varepsilon_{pitch,roll}=\sqrt{{\left(2\tan^{-1}\frac{\sigma_{CDGNSS,v}}{2r}\right)}^{2}+\sigma_{cam}^{2}} \end{array},$$

where *σ_cam_* is the standard deviation (STD) related to the uncertainty in the deputy detection and tracking process using a visual camera (which can be set equal to its instantaneous field of view, IFOV, assuming one pixel-level accuracy in deputy position estimation on the image plane [34]), whilst *σ_CDGNSS,h_* and *σ_CDGNSS,v_* are the horizontal and vertical STDs associated with CDGNSS processing, respectively, and *r* is the chief-to-deputy distance. The total expected pointing error (*ε*) is obtained by summing up the pitch, roll, and yaw contributions with

(13) $$\varepsilon =\sqrt{2{\varepsilon_{pitch,roll}}^{2}+{\varepsilon_{heading}}^{2}}.$$

**Figure 8:**
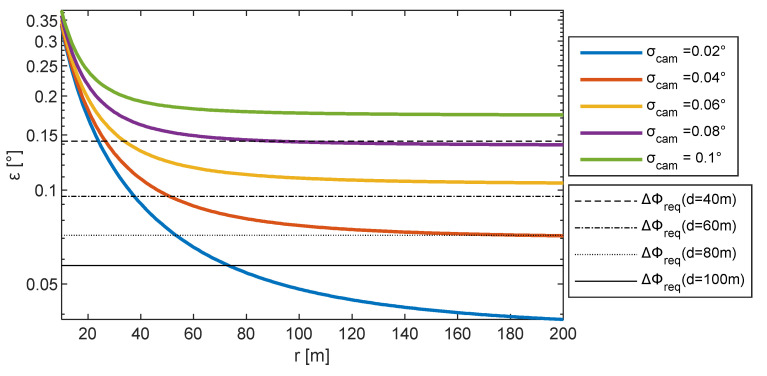
Required (ΔΦ*_req_*) and expected pointing error in CDGNSS/vision operation (*ε*). ΔΦ*_req_* has been estimated using Δ*x*_Φ_ = 0.1 m.

The expected pointing error using CDGNSS/visual techniques is reported in Figure 8, along with the value of ΔΦ*_req_* corresponding to different mapping scenarios in terms of operating distance and assuming that the maximum allowable Δ*x*_Φ_ is 10 cm. *ε* has been estimated by assuming *σ_CDGNSS,h_* = 0.02 m and *σ_CDGNSS,v_* = 0.04 m and varying *σ_cam_* from 0.02° to 0.1° which is typical for cameras embarked onboard UAVs. The figure shows how pointing accuracy decreases with range by reaching a plateau whose value depends on the camera IFOV. Only high-resolution cameras (with *σ_cam_* < 0.02°) allow the meeting of accuracy requirements when operating at 100 m distance from the object. The figure suggests choosing a large range to improve the attitude point accuracy. Nevertheless, the range should be compliant with the one ensuring air-to-air visual detection and tracking. Once the distance from the object to inspect is set, Figure 8 can be used to identify the value of range between the cooperative platforms, above which an attitude accuracy lower than the required one is ensured. However, such constraint can be relaxed if non-horizontal formations are exploited. Indeed, they allow more accurate pointing that the one predicted by Equation (13), as will be discussed in Section 6.

## 5. Attitude Error Prediction from Formation Geometry

### 5.1. Navigation Filter

**Figure 9:**
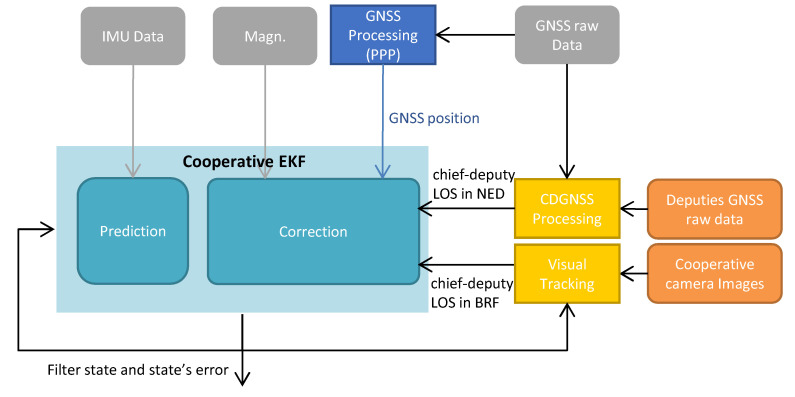
Block diagram of the cooperative EKF architecture. CDGNSS inputs are reported in yellow, GNSS processing converting raw data in positioning solutions are reported in blue.

The usage of the CDGNSS/visual process allows one to achieve an unbiased attitude estimate of the vehicle referred to as chief by exploiting two types of information, namely chief-to-deputy CDGNSS baseline in NED and camera-based line-of-sight in BRF. This information, retrieved at the GNSS sampling frequency, should be integrated within a filtering scheme for increasing the chief’s attitude reconstruction frequency to be used for payload data geolocation. Therefore, each vehicle equipped with mapping payload needs to act as a chief and to implement on-board an Extended Kalman Fiter (EKF), referred to as “cooperative EKF”, whose architecture is depicted in Figure 9. The EKF assumes the state is composed of the 3 × 1 vector, including the position **p** of the vehicle in geographic coordinates (i.e., latitude *l*, longitude *λ* and altitude *h*), the 3 × 1 velocity vector expressed in the NED frame, i.e., **v***^n^*, the 3 × 1 vector including the attitude angles from NED to BRF composed of the 321 sequence (yaw *ψ*, pitch *θ* and roll *φ*), and the 3 × 1 vectors including the accelerometer and gyroscope biases, expressed in BRF, i.e., $b_{a}^{b}$ and $b_{g}^{b}$ respectively. The filter propagates and corrects the state’s error *δ***x**, which is given by

(14) $$\delta x=\left[\begin{array}{c} \delta p\\ \delta v^{n}\\ \rho \\ \delta b_{a}^{b}\\ \delta b_{g}^{b} \end{array}\right];\begin{array}{c} \delta p={\left[\begin{array}{ccc} \delta l & \delta \lambda & \delta h \end{array}\right]}^{T}\\ \delta v^{n}={\left[\begin{array}{ccc} \delta v^{n}(n) & \delta v^{n}(e) & \delta v^{n}(d) \end{array}\right]}^{T}\\ \rho ={\left[\begin{array}{ccc} \rho (n) & \rho (e) & \rho (d) \end{array}\right]}^{T} \end{array},$$

where **ρ** represents the attitude error vector in NED [35]. 

The state and its error are propagated using IMU measurements with the well-known inertial navigation equations [35]. The correction step is entrusted to the classic Kalman equation, and the measurement vector includes GNSS positioning (yGNSS), magnetometer-based heading (yMAG), and CDGNSS/visual bearing information. Their associated measurement residuals are referred to as δyGNSS, δyMAG, and $\delta y_{j}={\left[\begin{array}{cc} \delta Az_{j} & \delta El_{j} \end{array}\right]}^{T}$. The latter is the CDGNSS/visual residual defined for the j-th deputy (j = 1, …, J). It is a 2 × 1 vector composed of Azimuth and Elevation residuals obtained by comparing the visual-based LOS estimate between the chief and the j-th deputy with the same quantity retrieved converting CDGNSS measurements in camera coordinates.

Measurement residuals are connected to the state error (*δ***x**) with the matrix *H*, as reported in Equation (15), which also includes the measurement covariance *R*. *R* is obtained by concatenating the GNSS, magnetometer, and CDGNSS/visual covariances referred to as *R_GNSS_*, *R_mag_,* and *R_j_* (for the *j*-th deputy), respectively.

(15) $$\begin{array}{c} \left[\begin{array}{c} \delta y_{GNSS}\\ \delta y_{MAG}\\ \delta y_{1}\\ \vdots \\ \delta y_{J} \end{array}\right]=\overset{H}{\overset{︷}{\left[\begin{array}{ccccc} H_{GNSS,p} & 0_{3\times 3} & 0_{3\times 3} & 0_{3\times 3} & 0_{3\times 3}\\ 0_{1\times 3} & 0_{1\times 3} & H_{MAG,\rho } & 0_{1\times 3} & 0_{1\times 3}\\ & & H_{1} & & \\ & & \vdots & & \\ & & H_{J} & & \end{array}\right]}}\left[\begin{array}{c} \delta p\\ \delta v^{n}\\ \rho \\ \delta b_{a}^{b}\\ \delta b_{g}^{b} \end{array}\right]\\ R=\left[\begin{array}{ccccc} R_{GNSS} & 0_{3\times 1} & 0_{3\times 2} & \cdots & 0_{3\times 2}\\ 0_{1\times 3} & R_{MAG} & 0_{1\times 2} & \cdots & 0_{1\times 2}\\ 0_{2\times 3} & 0_{2\times 1} & R_{1} & \cdots & 0_{2\times 2}\\ \vdots & \vdots & \vdots & \ddots & \vdots \\ 0_{2\times 3} & 0_{2\times 1} & 0_{2\times 2} & \cdots & R_{J} \end{array}\right] \end{array}.$$

0*_a_*_×*b*_ indicates a matrix composed of all zero elements with *a* rows and *b* columns. *H_a,_***_b_** is the matrix that connects the measurement *a* with the **b**-th part of the state. *H_GNSS,_***_p_** is an identity matrix of size 3 × 3 and *H_MAG,**ρ**_* = [0 0 1]. *H_j_* is the 2 × 15 measurement matrix associated with the *j*-th deputy. It is characterized by all zeros except for the elements connecting the cooperative residuals with the attitude part of the navigation state, which are incorporated in the matrix *H_j,_***_ρ_**:

(16) $$H_{j}=\left[\begin{array}{ccccc} 0_{2\times 3} & 0_{2\times 3} & H_{j,\rho } & 0_{2\times 3} & 0_{2\times 3} \end{array}\right].$$

The covariance matrix associated with the *j*-th deputy residual, i.e., *R_j_*, is a square matrix with size 2 × 2 equal to

(17) $$R_{j}=B_{j}{\left[\begin{array}{ccc} \sigma_{CDGNSS,h} & 0 & 0\\ 0 & \sigma_{CDGNSS,h} & 0\\ 0 & 0 & \sigma_{CDGNSS,v} \end{array}\right]}^{2}B_{j}^{T}+{\left[\begin{array}{cc} \sigma_{cam} & 0\\ 0 & \sigma_{cam} \end{array}\right]}^{2},$$

where *B_j_* is the matrix which maps the CDGNSS positioning error to *R_j_*. The formulation for δ**y***_j_*, *H_j**ρ**_*, and *B_j_* is not reported here for the sake of brevity, so the interested reader is referred to [36]. 

### 5.2. Attitude Error Prediction

The geometry of the chief/deputies’ formation affects both *H_j**ρ**_* and *B_j_* elements, shaping the contribution of the cooperative navigation information to the attitude aid. In GNSS-based applications, the impact of satellite geometry on the positioning solution is given by the Dilution of Precision (DOP) concept. In [26], the authors presented the generalized DOP (geDOP), which extends the DOP concept by including the contribution of the cooperative formation geometry to the positioning accuracy (which is of high interest in GNSS-challenging conditions) thanks to the knowledge of measurement and covariance matrices of the filter, *H*, and *R*, respectively. In the present paper, the geDOP concept is extended with the aim of providing a metric to link the attitude accuracy with the cooperative formation geometry. To this aim, only the columns of matrix *H* connected to attitude estimation should be used. Indeed, *H* does not contain any cross-coupling term among the different components of the state vector, namely δ**p**, δ**v**, and **ρ**, as shown in Equations (15) and (16). Therefore, the expected attitude uncertainties of the cooperative EKF in NED (**ε_ρ_**) and in BRF ($\varepsilon_{\rho_{b}}$) are

(18) $$\begin{array}{c} \varepsilon_{\rho }=\left[\begin{array}{c} \varepsilon_{\rho_{n1}}\\ \varepsilon_{\rho_{n2}}\\ \varepsilon_{\rho_{n3}} \end{array}\right]=\sqrt{diag{\left({\overset{⌣}{H}}^{T}{\left(\overset{⌣}{R}\right)}^{-1}\overset{⌣}{H}\right)}^{-1}}\\ \varepsilon_{\rho_{b}}=\left[\begin{array}{c} \varepsilon_{\rho_{b1}}\\ \varepsilon_{\rho_{b2}}\\ \varepsilon_{\rho_{b3}} \end{array}\right]=\sqrt{diag{\left(C_{n}^{b}{\overset{⌣}{H}}^{T}{\left(\overset{⌣}{R}\right)}^{-1}\overset{⌣}{H}{\left(C_{n}^{b}\right)}^{T}\right)}^{-1}}\\ \overset{⌣}{H}=\left[\begin{array}{c} H_{MAG,\rho }\\ H_{1\rho }\\ \vdots \\ H_{J\rho } \end{array}\right]=\left[\begin{array}{c} \begin{array}{ccc} 0 & 0 & 1 \end{array}\\ H_{1\rho }\\ \vdots \\ H_{J\rho } \end{array}\right];\overset{⌣}{R}=\left[\begin{array}{cccc} R_{MAG} & 0_{1\times 2} & \cdots & 0_{1\times 2}\\ 0_{2\times 1} & R_{1} & \cdots & 0_{2\times 2}\\ \vdots & \vdots & \ddots & \vdots \\ 0_{2\times 1} & 0_{2\times 2} & \cdots & R_{J} \end{array}\right] \end{array}.$$

$\varepsilon_{\rho_{ai}}$ is the component of the attitude accuracy along the *i*-th axis of the reference frame *a*, which could be either BRF (indicated with *b*) or NED (*n*). $C_{n}^{b}$ is the rotation matrix from NED to BRF and *diag*() is the operator extracting the diagonal of the matrix in the brackets. Expressing the attitude error in BRF allows easily connecting this information with heading, pitch, and roll angle. Indeed, when small pitch and roll angles are considered, which is often the case in a rotorcraft flight, the three components of **ε_ρ_***_b_* can be easily approximated to roll, pitch, and yaw angle uncertainty, respectively.

Equation (18) introduces for the first time an analytical formulation to derive the attitude angles, which were only numerically estimated by the authors in [37]. According to this equation, the attitude accuracy depends on *H_j**ρ**_* and on magnetometer and cooperative measurement covariance, i.e., *R_MAG_* and *R_j_*. As mentioned before, *H_j**ρ**_* and *R_j_* are functions of the relative geometry between the chief and the *j*-th deputy and of camera and CDGNSS processing specifications. Therefore, once the sensors are selected, **ε_ρ_***_b_* (and **ε_ρ_**) only depends on the formation geometry, which is optimal if it minimizes the norm of **ε_ρ_**, i.e., ‖**ε_ρ_**‖ = ‖**ε_ρ_***_b_*‖. Once this optimal formation has been identified, it must be kept for the whole flight duration to maximize the attitude accuracy.

## 6. Path Design for Multi-Drone Mapping

Taking advantage of the tools and methodologies introduced in Section 2, Section 3, Section 4 and Section 5, this section proposes a framework to plan inspection paths for multiple-UAV formations. As previously anticipated, path definition is aimed at improving mapping accuracy by both reducing the navigation error (to make attitude accuracy compliant with the required one) and increasing data collection capabilities. Hence, it consists of both solving the coverage problem and defining the formation geometry, which yields an attitude error compliant with the requirements. The relative formation among the platforms is assumed to be constant during the whole mission. A formation of three vehicles (i.e., two deputies and one chief) is assumed, which is the simplest configuration providing a fully observable state for the chief with parallel trajectories to the deputies, as demonstrated in [36]. 

**Figure 10:**
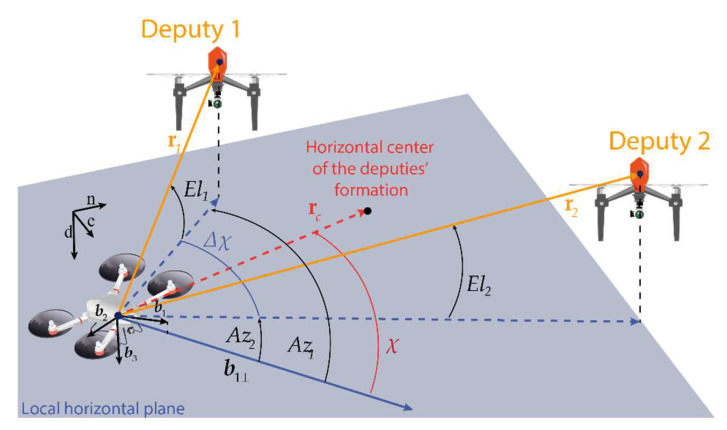
Cooperative formation geometry of a “one chief–two deputies” formation.

For the sake of concreteness, the vehicle triplets and the parameters defining the relative displacement among vehicles are shown in Figure 10. The position of the *j*-th deputy with respect to the chief is identified by three parameters, namely azimuth (*Az_j_*), elevation (*El_j_*), and range (*r_j_*). *El_j_* is the angle between the chief-to-deputy vector, i.e., **r***_j_*, and the local horizontal (north-east) plane; *Az_j_* is computed as the angle between the projection of **b***_1_*, i.e., **b***_1_*_⟂_, and **r***_j_* on that plane. Starting from these definitions, the formation geometry can be identified by the following angular parameters, whose formulation is reported in Equation (19):

- The azimuth of the formation (*χ*), i.e., the angle between **b***_1_*_⟂_, and the horizontal center of the deputies’ formation.
- The aperture angle (Δ*χ*), i.e., the angle between the projection of the deputies’ LOS on the horizontal plane.
- The average elevation (*μ*) and the elevation difference (Δ*μ*).
  (19) $$\begin{array}{l} \chi =\left(Az_{1}+Az_{2}\right)/2\\ \Delta \chi =\left\|Az_{1}-Az_{2}\right\|\\ \mu =\left(El_{1}+El_{2}\right)/2\\ \Delta \mu =\left\|El_{1}-El_{2}\right\| \end{array}.$$

As far as the range is concerned, formation geometries will be defined assuming chief–deputies’ distance is the same for each deputy, i.e., *r*_1_ = *r*_2_ = *r*.

In the case only one vehicle is operating as chief and is equipped with mapping payload, the coverage problem consists of solving the CCP for the chief, while the deputy trajectories can be obtained by offsetting the chief’s path with the relative geometry, which minimizes the attitude error. The definition of the best relative geometry is given in Section 6.1. Otherwise, when each UAV is equipped with a mapping payload, relative formation definition and C-CCP is carried out altogether, while complying with both the attitude and the point-density requirement, as will be discussed in Section 6.2. In both cases, the process of relative formation geometry selection is composed of two steps:

1. Selecting the chief-to-deputy range required to attain the desired pointing error, i.e., ΔΦ*_req_*, as explained in Section 4.
2. Selecting the formation geometry’s characteristic angles (i.e., *χ*, Δ*χ*, *μ*, and Δ*μ*), which are compliant with the requirements (i.e., attitude, mapping payload, and cooperative camera FOV) and minimize the pointing error, as reported either in Section 6.1 for cooperative navigation only or in Section 6.2 for simultaneous cooperative navigation and data collection.

For the sake of concreteness, assuming *d* = 60 m, to satisfy point 1 above and achieve a value of Δ*x*_Φ_ = 0.1 m, a range at least equal to 60 m is needed if cooperative sensor equipment with *σ_cam_* = 0.04°, *σ_CDGNSS,h_* = 0.02 m, and *σ_CDGNSS,v_* = 0.04 m is used. This results in a value of attitude error predicted with Equation (13), i.e., ε_60_, equal to 0.089°. The formation geometry definition discussed in the following uses the same sensor performance reported above and assumes *r* = 60 m. In addition, ε_60_ is used as the desired attitude accuracy requirement.

### 6.1. Single-Chief Aircraft

This section aims at defining the formation geometry that minimizes the attitude error for only one aircraft of the formation (namely, the chief), which is in charge of the mapping mission. Since *r* is fixed by the pointing requirement, the formation geometry parameters to be analyzed are *χ*, Δ*χ*, *μ,* and Δ*μ*. 

**Figure 11:**
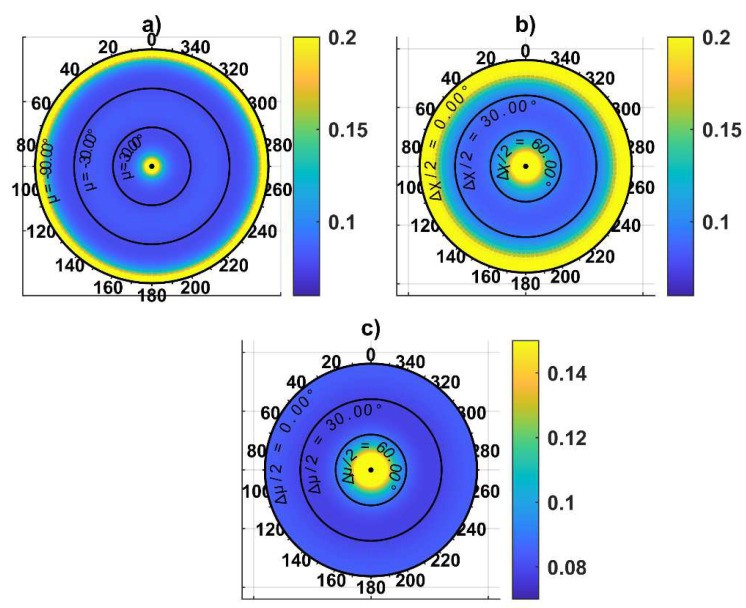
‖ε_ρ_‖ in degrees as a function of *χ* along the polar direction and (**a**) *μ* (Δ*χ* = 90°, Δ*μ =* 0°), (**b**) Δ*χ* (*μ =* Δ*μ =* 0°), and (**c**) Δ*μ* (Δ*χ* = 90°, *μ =* 0°) on the radial direction.

**Figure 12:**
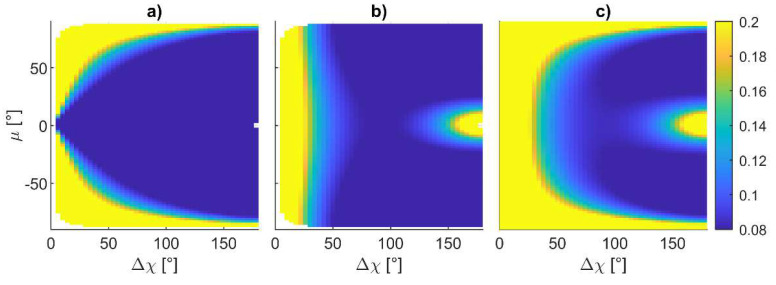
Attitude error in degrees as a function of Δ*χ* and *μ* and assuming Δ*μ* = 0°, (**a**) $\varepsilon_{\rho_{b3}}$, (**b**) $\sqrt{\varepsilon_{\rho_{b1}}^{2}+\varepsilon_{\rho_{b2}}^{2}}$, (**c**) ‖ε_ρ_‖.

Figure 11 shows the variation in ‖**ε_ρ_**‖ estimated with Equation (18) as a function of *χ* along the polar direction and Δ*χ*, *μ,* and Δ*μ* along the radial direction, respectively, in subfigures a to c, showing that the norm of the attitude error is independent from *χ*. This result implies that the vector connecting the vehicle with the center of the horizontal formation, which is indicated as **r***_c_* in Figure 10, can be pointed to any direction on the horizontal plane. The optimal value for the other parameters should be searched in a 3D space, where *μ* is varied between −90° and 90°, while Δ*μ* and Δ*χ* are varied between 0° and 180°. A first analysis has been performed by considering the value of the attitude error over the Δ*χ*-*μ* plane, with Δ*μ* = 0°, as reported in Figure 12. 

**Figure 13:**
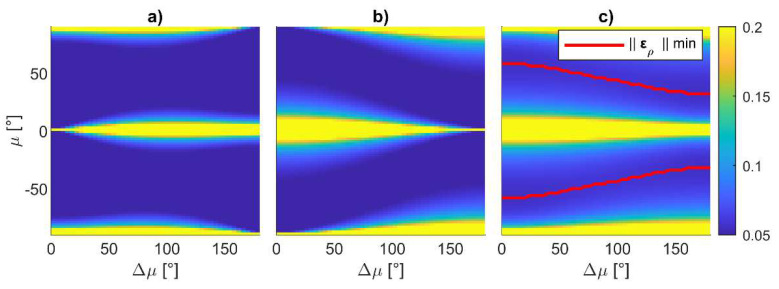
Attitude error in degrees as a function of Δ*μ* and *μ* and assuming Δ*χ* = 180°, (**a**) $\varepsilon_{\rho_{b3}}$, (**b**) $\sqrt{\varepsilon_{\rho_{b1}}^{2}+\varepsilon_{\rho_{b2}}^{2}}$, (**c**) ‖ε_ρ_‖, red lines indicate the locus of point where the norm of the attitude assumes a minimum value.

Specifically, Figure 12a,b show the heading and the combined pitch and roll errors, whilst Figure 12c reports the norm of the attitude error. The figure demonstrates that the angular error is symmetric with respect to elevation. Positive elevations are encouraged to avoid the deputies entering the FOV of the nadir-looking LiDAR when a horizontal surface must be inspected. The minimum attitude error (equal to 0.056° for the set of inputs used in this discussion) can be retrieved from Figure 12c, corresponding to a Δ*χ* = 180° and *μ =* 55°. This value is smaller than ε_60_, highlighting the advantage of tailoring the geometry of the cooperative formation. Fixing Δ*χ* = 180° and making Δ*μ* vary allows one to obtain the same minimum value by properly selecting *μ*. The combination of Δ*μ* and *μ* where the minimum value is obtained is identified by the red curve in Figure 13c, along with the norm of attitude error on the Δ*μ–μ* plane obtained assuming Δ*χ* = 180°. For the sake of completeness, the heading and the combined pitch and roll angles are reported in Figure 13a and Figure 13b, respectively. Figure 13 shows that several geometries minimizing the attitude error exist for the three UAVs. Those formations require the two vehicles belonging to the plane to be either orthogonal to **r***_c_* or contain the down unit vector and **r***_c_*. With the aim of keeping the formation symmetrical, the best single-chief formation can be obtained by setting Δ*μ* = 0°, Δ*χ* = 180°, and *μ =* 55°. In the horizontal surface inspection case, one can select *χ* equal to zero aligning the center of the formation with the forward direction of motion, thus making the plane on which the UAVs are contained orthogonal to that direction. Therefore, the deputy vehicles are positioned on the two sides of the chief trajectory during the mission. If a lateral surface must be inspected, the chief vehicle has on its side the object to inspect; therefore, the formation with *χ* = 0° potentially leads to a collision between the object and one of the two deputies. In this case, the formation must be rotated, setting *χ* = 90°. In both cases, in order to have the two deputies in the chief camera field of view, a zenith-looking visual system should be envisaged, with an FOV at least equal to 70°, i.e., 2·(90° − *μ*).

#### 6.1.1. Quasi-Horizontal Formations

The above-defined geometry is characterized by a high elevation angle, which makes the altitude of the deputies far higher than the chief one, especially when a high value of *r* is needed. In order to keep the vertical separation of the vehicles small, a quasi-horizontal geometry could be considered. This geometry is also compliant with the common forward-looking camera installations onboard the UAV. In this configuration, in order to select Δ*χ*, one must account for horizontal FOV limits, whilst the camera tilt angle can be selected equal to *μ*. 

**Figure 14:**
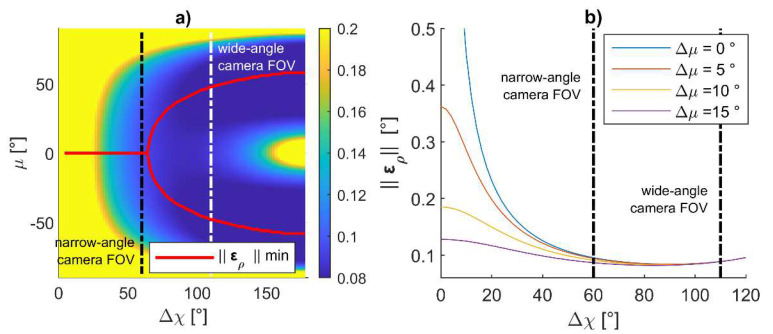
Selection of cooperative formation in horizontal camera configuration. (**a**) Norm of the attitude error in degrees as a function of Δ*χ* and *μ* and assuming Δ*μ* = 0°, red curves indicate the locus of point where the norm of the attitude error assumes a minimum value for each Δ*χ*, (**b**) norm of the attitude error as a function of Δ*χ* and Δ*μ* and assuming *μ* = 0°. Vertical lines indicate narrow and wide-angle FOV.

Following this approach, *μ* can be selected as the one minimizing the attitude error on the Δ*χ*–*μ* plane with Δ*μ* = 0°. Figure 14a reports a zoom on Figure 12c, where the FOV limits for a narrow angle (defined to have a FOV < 60°) and wide-angle camera (FOV ranging from 60° to 110°) are identified, along with the locus of points where the pointing angle assumes a minimum value for each Δ*χ*. When FOV < 60°, *μ* should be selected equal to zero. Conversely, the value of the optimal *μ* increases with Δ*χ* for wide-angle cameras. Nevertheless, in view of the constraint of limiting the vertical separation between vehicles, also for FOV > 60°, it is convenient to keep *μ* and the camera tilt angle equal to 0°. With this assumption, one can pick the formation which minimizes ‖**ε_ρ_**‖ and allows both the deputies to be in the chief’s FOV by selecting the adequate value of Δ*μ* and Δ*χ*. Figure 14b reports the value of ‖**ε_ρ_**‖ while varying Δ*χ*, and for several values of Δ*μ*, when *μ* = 0°. In order to keep the altitude variation small (and also taking vertical FOV limitations into account), the maximum value of Δ*μ* is selected as 15°. The impact of Δ*μ* is negligible when a horizontal angular separation, i.e., Δ*χ*, larger than 60° is chosen. 

It could be noticed that ‖**ε_ρ_**‖ assumes a minimum (‖**ε_ρ_**‖ = 0.084°) when Δ*χ* = 90°, which can be only obtained with a wide-angle camera. The minimum value for ‖**ε_ρ_**‖ is smaller than the expected error derived through Equation (13), i.e., ε_60_, suggesting the formation can effectively support the mapping missions. Conversely, when reducing Δ*χ* as required for narrow-angle cameras, the pointing accuracy becomes worse than ε_60_. To keep the attitude error compliant with the requirements, the range must be increased. 

In this case, a different procedure can be applied for geometry selection. Instead of defining range as reported in point 1 of the relative formation geometry definition procedure described above, one can set a formation which is compliant with the camera FOV and derive the range accordingly. Knowing that in the horizontal formation the attitude error decreases for increasing Δ*χ* (as reported in Figure 14), one can select the highest Δ*χ*, allowing the deputies to enter the FOV (e.g., it could be selected as 80% of the horizontal FOV). In addition, Δ*μ* and *μ* should be set equal to zero to prevent the far range from producing a huge vertical separation among the UAVs. 

Under these hypotheses, the required value for the range must be selected by predicting when the value of ‖**ε_ρ_**‖ becomes smaller than ΔΦ*_req_*. Nevertheless, the derivation of ‖**ε_ρ_**‖ with Equation (18) requires the inversion of $\overset{⌣}{R}$ and $\overset{⌣}{H}$, which can be complex and computationally heavy. In the case Δ*μ* = *μ* = *χ* = 0°, an explicit analytical expression can be derived connecting Δ*χ* and *r* with ‖**ε_ρ_**‖, which generalizes Equation (13) in the case Δ*χ* ≠ 90°.

(20) $$\left\|\varepsilon_{\rho }\right\|=\sqrt{2{\left(\frac{\varepsilon_{pitch,roll}}{\sin\Delta \chi }\right)}^{2}+{\varepsilon_{heading}}^{2}}.$$

**Figure 15:**
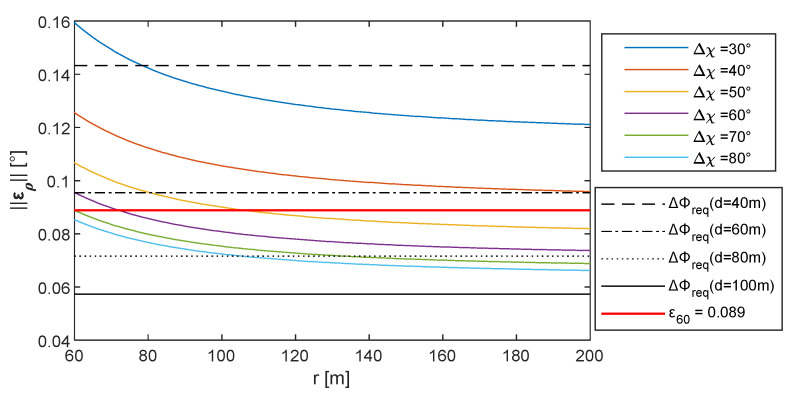
‖ε_ρ_‖ a function of range assuming Δ*μ* = *μ* = 0° and several values for Δ*χ*.

‖**ε_ρ_**‖ values obtained by varying ranges and by setting Δ*μ* = *μ* = 0° are reported in Figure 15, along with the required attitude error at different distances from the object and the line highlighting ε_60_. Apertures smaller than 50° do not enable one to obtain an attitude error smaller than ε_60_. Hence, a 50° aperture, which can be easily attained with the common camera lenses on board UAVs, allows one to retrieve a value of the accuracy angle compliant with ε_60_, selecting a range slightly higher than 100 m. Apertures greater than 60° allow one to obtain a value of attitude error compliant with ε_60_, also without increasing *r*. 

When horizontal formations (i.e., *μ* = 0°) are selected, in the case where the chief has to map a lateral surface, to prevent collision, the **r***_c_* direction can be selected to be orthogonal to **b***_1_*_⟂_ and pointing in the opposite direction with respect to the LiDAR (which is directed toward the building). Conversely, when nadir=looking missions have to be carried out, *χ* can be selected arbitrarily. However, a value of *χ* = 90° reduces the delays needed to wait the deputy to be placed in the correct position with respect to the chief in two subsequent lines of the zig-zag path.

### 6.2. Formation Design for Multiple-Chief Aircraft Platforms 

This section considers each vehicle of the formation has to collect LiDAR points, and mapping and precise navigation capabilities are required by all of them. In this case, the cooperative EKF described in 5.1 runs on each vehicle of the formation which simultaneously assumes chief and deputy functionality. The relative formation can be found by minimizing the sum of the attitude errors of each vehicle. In the following, each vehicle can be identified with a pedex *j* = 1, 2, 3. Therefore, the attitude error norm associated with each vehicle will be ‖**ε_ρ_**‖*_j_*, and the quadratic sum of these norms corresponding to the overall attitude error will be indicated as ‖**E_ρ_**‖. In order to use the previously defined parameters for identifying the formation geometry, *χ*, Δ*χ*, *μ,* and Δ*μ* will be identified, assuming vehicle 3 is occupying the chief location in Figure 10. As in the previous case, it could be shown that optimal formations are to be searched either on the plane orthogonal to **r***_c_* or on the plane where **r***_c_* and the down direction lay. However, when mapping capabilities are requested, the available geometries are limited by the scenario that has to be inspected.

**Figure 16:**
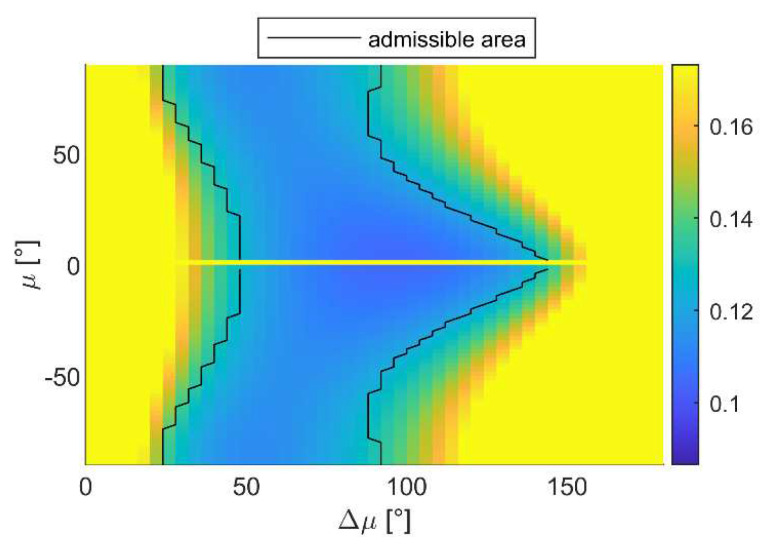
‖E_ρ_‖ in degrees as a function of Δ*μ* and *μ*, assuming Δ*χ* = *χ* = 0°. The area delimited by black lines (i.e., admissible area) identifies the points where ‖ε_ρ_‖*_j_* < ε_60_ ∀ *j*.

In the case of lateral surface mapping, the inspection path is characterized by parallel lines spaced along the vertical direction by Δ*s*. To increase the coverage capability, the UAVs of the formation should lie on subsequent lines at a constant distance from the object to inspect. Under these hypotheses, the optimal cooperative formation can be searched on the **b***_1_*_⟂_-down plane, assuming both *χ* and Δ*χ* are equal to zero. Figure 16 reports the value of ‖**E_ρ_**‖ as a function of the other two parameters that are allowed to vary, i.e., *μ* and Δ*μ*, highlighting that the best geometries are those which have a small *μ* (but different from zero), with Δ*μ* around 90°. All the combinations of *μ* and Δ*μ* included in the admissible area provide a value of ‖**ε_ρ_**‖*_j_* < ε_60_ ∀ *j* and can be selected as candidates. To keep the geometry symmetrical, a small value of *μ* can be set; hence, the value of Δ*μ* can be selected so that it belongs to the admissible area and is smaller than 2sin^−1^(Δ*s*/*r*), to ensure footprints overlap. The so-chosen formation enables the movement of the three UAVs on parallel lines, with UAV 1 and UAV 2 preceding UAV 3 during the motion, of a length equal to *r*cos(Δ*μ*/2).

**Figure 17:**
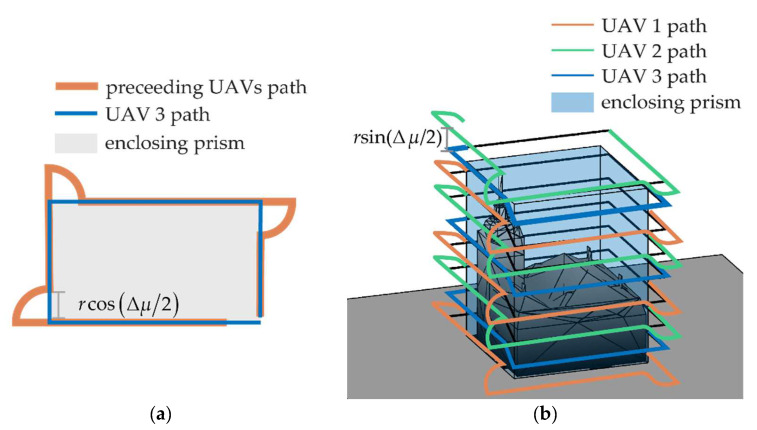
Path of the cooperative formation for lateral face inspection, (**a**) top and (**b**) 3D view.

As shown in Figure 17b, the inspection pattern around an object enclosed on a generic prism can be obtained by increasing the altitude of the vehicle(s) once they have completed an entire perimeter of the enclosing prism. The top view of the perimeter coverage of a prism with a rectangle shape is reported in Figure 17a for a formation of three vehicles. Since *μ* is set very close to zero, the trajectories of the preceding vehicles are overlapping on the top view representation. At the end of each side of the perimeter. the preceding vehicles have to cover an arc of a circumference with a radius *r*cos(Δ*μ*/2), while UAV 3 rotates its heading in order to keep them in its FOV. 

To map a horizontal surface, quasi-horizontal formation geometries are suggested (small values of *μ* and Δ*μ*). Indeed, high values of *μ* and Δ*μ* could produce an increased distance from the surface to inspect, which limits the point density and could potentially bring the platform to altitudes higher than the maximum range of the LiDAR. Therefore, in line with Section 6.1.1, *μ* is set equal to zero and Δ*μ* is varied between 0 and Δ*μ_max_*, which is the value that would produce a decrease in point density which is below a certain percentage, i.e., Δ*ω*. Once *r* and *d* are known and since the point density (i.e., *ω*) is proportional to 1/*d*, one can write

(21) $$\Delta \mu_{\max}=\sin^{-1}\left(\frac{d\Delta \omega }{\left(1-\Delta \omega \right)r}\right).$$

**Figure 18:**
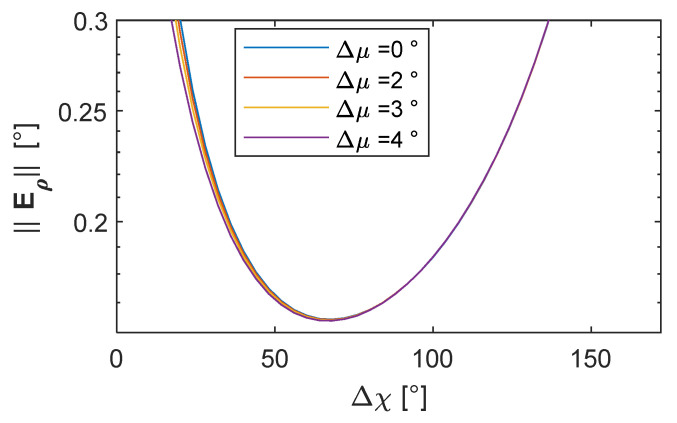
‖E_ρ_‖ as a function of Δ*χ* and varying Δ*μ* between 0° and Δ*μ_max_*, *μ* = 0°.

**Figure 19:**
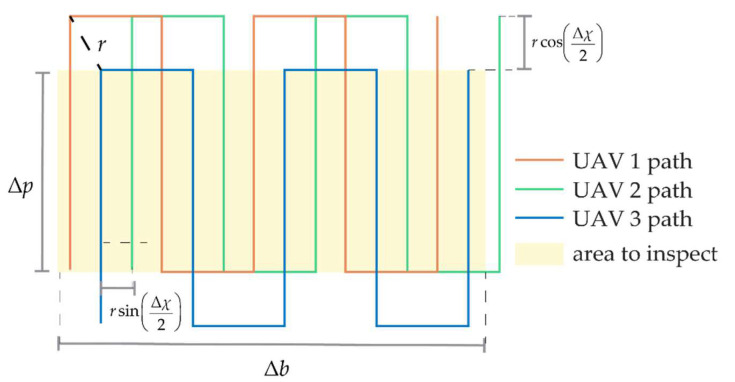
Path of the three UAVs to map a horizontal area, top view.

Figure 18 reports several combinations of Δ*χ* and Δ*μ* and their associated ‖**E_ρ_**‖ value using *r* = 60. The optimal Δ*χ* value corresponds to 60°, suggesting the placement of the vehicles in the vertex of an equilateral triangle. Only wide-angle cameras are used onboard the three UAVs, ensuring this formation is realized, which results in ‖**ε_ρ_**‖*_j_* = 0.095° (in case *μ* = 0°). This value is slightly higher than ε_60_, thus requiring one to apply a range increase strategy analogous to the one described in Section 6.1.1. Also in this case, the formation assumes UAV 1 and UAV 2 preceding UAV 3 of *r*cos(Δ*χ*/2); therefore, for a complete coverage of a strip of length Δ*p*, the trajectory length of each vehicle over that strip should be equal to Δ*p + r*cos(Δ*χ*/2). The horizontal spacing between the platform is equal to *r*sin(Δ*χ*/2), where Δ*χ* can be at most equal to 60°, in the optimal formation case. When *r*sin(Δ*χ*/2) < Δ*s*, one can increase *r* to be compliant with Δ*s*, which also produces an advantage in terms of cooperative navigation. However, the increase in *r* could stretch the trajectory length over Δ*p*. Over a rectangle of size Δ*p*×Δ*b* (with Δ*p* being the main direction of motion), the total trajectory length for each UAV is equal to

(22) $$\left(\Delta p+r\cos\left(\frac{\Delta \chi }{2}\right)\right)\left\lceil \frac{\Delta b}{n_{u}r\sin\left(\frac{\Delta \chi }{2}\right)}\right\rceil +\left(\left\lceil \frac{\Delta b}{n_{u}r\sin\left(\frac{\Delta \chi }{2}\right)}\right\rceil -1\right)n_{u}r\sin\left(\frac{\Delta \chi }{2}\right).$$

where *n_u_* represents the UAV number and ⌈⌉ is the ceiling operator of the quotient’s result. For the sake of clarity, the paths of the vehicles and the quantities accounted for in Equation (22) are reported in Figure 19.

## 7. Simulation Results

**Figure 20:**
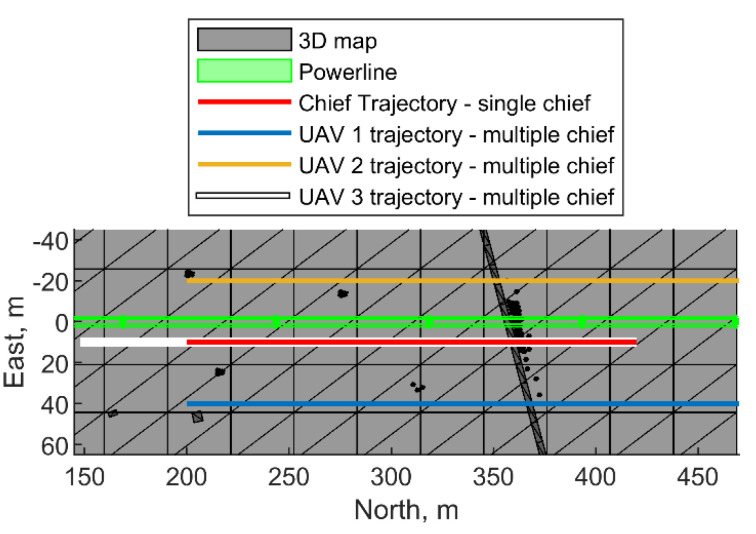
Powerline inspection scenario, top view. Powerline structure is highlighted with green color. Trajectory of UAVs in case of single-chief (red) and multiple-chief (white, blue, yellow) cases is also reported.

In this section, the trajectory design of the cooperative formation presented in the previous sections is tested in a simulation framework considering the powerline inspection scenario reported in Figure 20. A simple scenario which does not require a zig-zag path has been used to put emphasis on the definition of the formation geometry. The formation has to inspect the powerline segment spanning from 200 m to 420 m in the North direction. A simulation has been carried out in MATLAB by using the LiDAR toolbox for retrieving the points in the LiDAR reference frame (LRF). Both trajectory reconstruction and LiDAR data georeferencing performance are assessed in this section. Navigation accuracy in terms of positioning and attitude errors is obtained by comparing the values estimated with the cooperative EKF with the simulated “true” ones. Conversely, georeferencing accuracy is obtained by comparing the true point clouds with the ones obtained using the EKF-based trajectory. The mapping vehicle(s) is/are assumed to embark a nadir-looking Velodyne Puck LiDAR and the Honeywell HG1120BA50 [38] IMU, whose parameters are reported in Table 2. CDGNSS/visual processing is simulated by assuming *σ_cam_* = 0.04°, *σ_CDGNSS,h_* = 0.02 m, and *σ_CDGNSS,v_* = 0.04 m. The simulation of GNSS measurements assumes a decimeter-level mean and a centimeter-level standard deviation, as expected by typical PPP processing performance. A formation of three vehicles is considered and the desired georeferencing accuracy due to attitude, i.e., Δ*x*_Φ_, is set equal to 0.1 m. To keep the point density high while guaranteeing a separation from the powerline (which is 35 m tall) to prevent magnetic interference, the minimum altitude of the formation is assumed to be 60 m.

### 7.1. Cooperative Formation—Single Chief

In the case where there is only one vehicle in charge of the mapping task, its trajectory is selected as parallel to the powerline at an altitude equal to the minimum altitude of the formation, i.e., 60 m. The trajectory, depicted in red in Figure 20, spans from 200 m to 420 m along the north axis to cover the required segment, with Δ*p* = 220 m. Its velocity should be tailored to have an acceptable point density. To this aim, a 1 m/s velocity, which is commonly used for mapping applications, is set, resulting in a 736-points/m^2^ point density, as predicted with Equation (5). Cooperative formation geometry foresees the definition of *r*, *χ*, Δ*χ*, *μ*, and Δ*μ.* Since powerline inspection consists of nadiral mapping, *χ =* 0°. Two different cooperation camera FOVs are accounted for in this discussion, which lead to two different formations, as reported in Section 6.1, i.e., as follows:

Formation 1: If a wide FOV camera is used, the distance among the elements of the formation can be obtained by using Equations (11) and (13) and Figure 8. The range to attain the required pointing accuracy when *d* = 60 m and using the above-reported CDGNSS and camera STDs is about 60 m. The best formation for the cooperative geometry foresees Δ*χ* = 180°, Δ*μ* = 0°, and *μ* = 55°.
Formation 2: When a limited FOV is available (i.e., equal to 64° in the following) and if a very small vertical separation between the vehicles of the formation is needed following the strategy reported in Section 6.1.1, one can obtain, Δ*μ* = *μ* = 0° and Δ*χ* = 50°. Hence, the distance among the vehicles of the formation can be obtained equating Equations (11) and (20), which results in *r* = 85 m.

**Figure 21:**
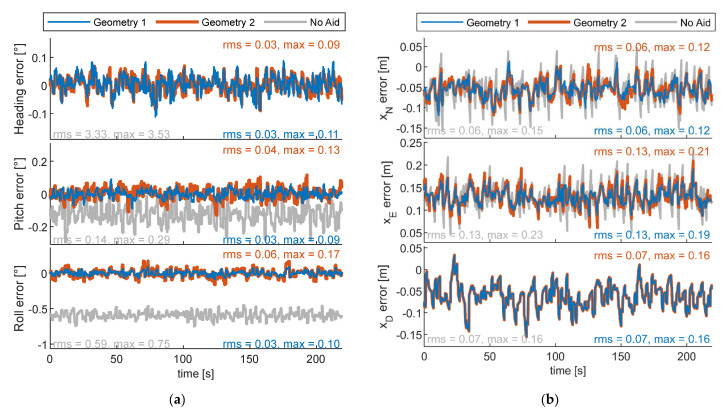
Navigation result of the chief UAV with formation 1 or 2, or no cooperation. One-chief case. (**a**) Attitude and (**b**) positioning errors.

The attitude and positioning errors along the trajectory achieved by the chief vehicle using either formation 1 or 2 are reported in Figure 21. These results are compared with those obtained in the case where no cooperation is used, i.e., the filter described in Figure 9 and Section 5.1 does not use cooperative inputs. Positioning errors are reported in Figure 21b. Cooperation allows one to reduce the east and north errors, showing only a slight difference in performance between the two geometries. Nevertheless, due to a slightly biased GNSS error, filter results in terms of positioning appear to have an offset with respect to the true value which is equal to [−0.05 0.13 −0.06] in NED. Figure 21a shows attitude errors and their associated root mean square (RMS) and maximum value. Cooperation allows one to reduce the heading error to the sub-degree level. Without cooperation, the heading error is biased because of the magnetometer estimate, reaching a mean value of −3.32°. For the sake of visualization, it has not been reported in the figure. The norm of the RMS error obtained using geometry 1 (i.e., 0.052°) is smaller than the case where geometry 2 is used and complaint with the expected value, retrieved from Figure 12. Geometry 2, which has been selected with the procedure reported in Section 6.1.1, allows one to obtain, as expected, a pointing error which is smaller than the required one and is equal to 0.078°. It is important to remark that the attitude accuracy also depends also on the IMU, which can reduce the error with respect to the one predicted with Equation (18). In fact, the latter equation does not account for the IMU performance level and defines an upper bound for the attitude error [26].

**Figure 22:**
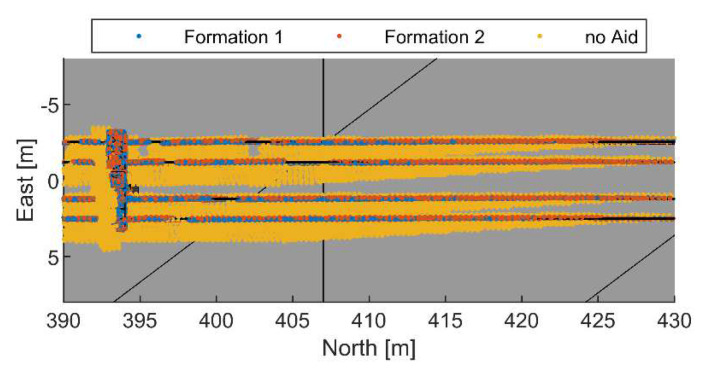
Zoom on the reconstructed powerline with formation 1 or 2, or no cooperation. Single-chief case.

The georeferencing accuracy is reported in Table 3, by considering four sets of data which have been obtained after segmenting the point cloud, in Full Set (which corresponds to the whole point cloud), Lines, Pillars, and Powerline (which includes both lines and pillars). Scene segmentation has been carried out with the procedure reported in [39]. The biased heading in the no-cooperation case causes a significant georeferencing error (in the order of meters), which is not compliant with the requirements. Conversely, the use of cooperation allows one to attain the centimeter-level RMS in georeferencing products for both formation 1 and formation 2. Due to the higher pointing error, the georeferencing performance is slightly worse for formation 2, leading to an overall value of 17.6 cm when the full set is considered. A zoomed view of the georeferenced point cloud including only the points belonging to the powerline and excluding the terrain is shown in Figure 22. Points obtained with the three georeferenced results are shown. When cooperation is not used, the quality of the final mapping product is degraded, as highlighted by the thicker lines identified by the yellow points. Conversely, a very accurate product (with points correctly placed on the “true” lines) is obtained in the cooperative case. The obtained point density estimated in area of 10 × 10 m at the center of the trajectory covered by the UAV is equal to 706 points/m^2^, which is slightly smaller than the predicted one.

### 7.2. Cooperative Formation for Multiple-Chief Aircraft

**Figure 23:**
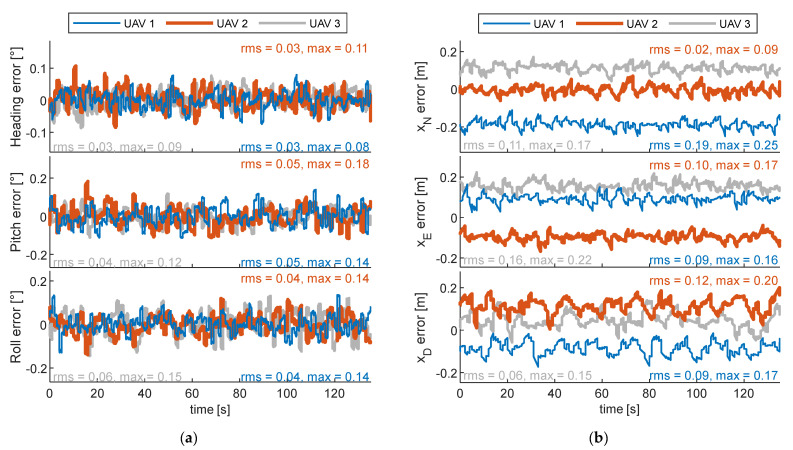
Navigation result of the UAVs 1 to 3, demanded simultaneously at mapping and cooperation aid. (**a**) Attitude and (**b**) positioning errors.

The formation geometry demanded at simultaneously optimizing the LiDAR data collection and georeferencing performance consists of an equilateral triangle (*μ* = Δ*μ* = *χ*= 0°, Δ*χ* = 60°) where UAVs are kept at constant altitude. The altitude of the formation is set equal to 60 m; hence, *r* = 60 m is retrieved from Figure 8. UAVs 1 and 2 precede UAV 3 in the formation, whose top-view is also reported in Figure 20. The formation is not symmetrical with respect to the powerline to avoid the fact that a vehicle fault could damage the structure. Specifically, UAV 3 moves on the white path reported in Figure 20. It overlaps with the one describing the chief trajectory in Section 7.1, (reported with red in Figure 20) but it has a longer path length, set equal to Δ*p + r*cos(Δ*χ*/2) to enable uniform coverage of the area to inspect. To this aim, the starting north coordinate of UAV 3 has been pushed backward with respect to the red line and is equal to 148 m, resulting in a path length equal to 272 m, until the last point, whose north coordinate is again equal to 420 m. The spacing between UAV 1 and 2 with respect to UAV 3 in the direction orthogonal to the motion (i.e., the East direction), equal to Δ*s = r*sin(Δ*χ*/2) = 30 m, is far smaller than the LiDAR footprint Δ*l* (equal to 160 m), thus potentially enabling the velocity to increase. Specifically, it can be set to 2 m/s, leading to a reduction in the mission time (135 s, instead of 220 s), despite the trajectory length increase with respect to Section 7.1. The navigation solution for the three UAVs is reported in Figure 23. The equilateral geometry returns a similar attitude performance of the three vehicles, whose pointing accuracy is always better than the required one. Conversely, the positioning performance highlights decimeter-level biases for each platform. The georeferencing accuracy is summarized in Table 4, resulting in an overall error of 20.4 cm in the full set case. The total point density is 1017 points/m^2^, which is higher than the one obtained in the previous section despite the velocity increase, highlighting the advantage of cooperative mapping.

## 8. Conclusions

This paper analyzed mission planning and cooperative navigation and mapping algorithms for multi-drone-based LiDAR surveys, considering a three-aircraft system. Cooperation can be exploited to improve attitude estimation and thus LiDAR data georeferencing accuracy, and/or to improve coverage per unit time. In both frameworks, analytical relations have been derived to support centralized end-to-end mission planning. 

As concerns LiDAR coverage, an analytical formulation for point cloud density estimation has been derived for both omnidirectional and non-repetitive scanners, and trajectory planning approaches to solve the CCP problem have been defined, also shaping the formation to the navigation needs. 

As far as navigation performance is concerned, the CDGNSS/Vision cooperative navigation paradigm has been used to obtain fine attitude accuracy. This paper extended the generalized dilution of precision concept to the attitude estimate, thus providing a metric for formation geometry optimization. Attitude error minimization can be achieved using a large spatial and angular distance among vehicles (i.e., 180° and 60° horizontal separation if one or all the vehicles are used for the mapping task, respectively), thus imposing the use of a wide FOV visual system. Formation optimization in the case a narrow FOV visual system has also been tackled, suggesting quasi-horizontal formations should be preferred. An analytical formulation was also derived to link the required georeferencing accuracy and the attitude estimation performance. 

The developed concepts and relations have been tested in a simulated scenario involving powerline inspection. The expected attitude error matches their higher bound predicted during formation design, showing very accurate georeferencing results when cooperation is used. Indeed, an order of magnitude improvement in georeferencing error results from using a cooperative approach.

Future works area aimed at building an experimental setup to demonstrate the applicability of the proposed methodology in real-world scenarios and to further indicate the advantage of using cooperation among UAVs.

## Figures and Tables

**Table 2:** Simulated IMU parameters.

Accelerometer			Gyroscope		
Accel Bias Repeatability (mg)	Accel Bias In-run Stability (mg)	Velocity random walk (m/s/√h)	Gyro Bias Repeatability (°/h)	Gyro Bias In-run Stability (°/h)	Angular Random Walk (°/√h)
10	0.05	0.06	520	24	0.4

**Table 3:** Georeferencing results. One chief.

*Scene* *Component*	Metric	LiDAR Data Georeferencing Accuracy, cm								
		Formation 1			Formation 2			No Aid		
		N	E	D	N	E	D	N	E	D
Full	RMS	6.5	13.6	7.0	6.5	14.6	7.3	162.5	98.2	26.7
	MAX	20.2	25.1	18.7	21.3	31.2	28.9	395.8	240.0	79.4
Powerline	RMS	6.3	13.5	6.9	6.3	14.2	7.0	102.4	86.3	13.5
	MAX	17.9	22.9	17.3	19.6	29.5	18.8	239.5	201.5	41.4
Lines	RMS	6.2	13.5	6.8	6.2	14.2	6.9	73.7	85.4	5.8
	MAX	15.8	23.0	16.1	18.7	28.5	18.4	107.6	173.3	19.6
Pillars	RMS	6.4	13.4	6.9	6.5	14.2	7.1	142.3	201.5	21.7
	MAX	17.9	22.5	17.3	19.6	29.5	18.8	239.5	201.5	41.4

**Table 4:** Georeferencing results. The three vehicles simultaneously assume chief and deputy roles.

Metric	Georeferencing Accuracy, cm											
	Full			Powerline			Lines			Pillars		
	N	E	D	N	E	D	N	E	D	N	E	D
RMS	12.8	12.8	9.5	13.2	12.3	9.4	13.1	12.5	9.3	13.6	11.6	9.7
MAX	35.0	31.5	33.4	33.2	29.3	25.3	31.6	28.7	25.3	33.2	29.3	22.1

## Data Availability

Data are contained within the article.
